# Crystal structure of ammonium bis­[(pyridin-2-yl)meth­yl]ammonium dichloride

**DOI:** 10.1107/S2056989015015753

**Published:** 2015-08-29

**Authors:** Aaron Trischler, Kayode Oshin, Tomislav Pintauer

**Affiliations:** a Department of Chemistry & Physics, Saint Marys College, Notre Dame, IN 46556, USA; b Department of Chemistry and Biochemistry, Duquesne University, Pittsburgh, PA 15282, USA

**Keywords:** crystal structure, protonated structure, hydrogen bonding, atom transfer radical addition (ATRA) reactions, chirality

## Abstract

In the title molecular salt, C_12_H_14_N_3_^+^·NH_4_^+^·2Cl^−^, the central, secondary-amine, N atom is protonated. The bis­[(pyridin-2-yl)meth­yl]ammonium and ammonium cations both lie across a twofold rotation axis. The dihedral angles between the planes of the pyridine rings is 68.43 (8)°. In the crystal, N—H⋯N and N—H⋯Cl hydrogen bonds link the components of the structure, forming a two-dimensional network parallel to (010). In addition, weak C—H⋯Cl hydrogen bonds exist within the two-dimensional network.

## Related literature   

For background to atom-transfer radical addition reactions, see: Eckenhoff & Pintauer (2010 ▸); Kharasch *et al.* (1945 ▸); Iqbal *et al.* (1994 ▸); Braunecker & Matyjaszewski (2007 ▸); Matyjaszewski *et al.* (2001 ▸); Tang *et al.* (2008 ▸). For the synthesis, see: Carvalho *et al.* (2006 ▸). For related structures, see: Junk *et al.* (2006 ▸).
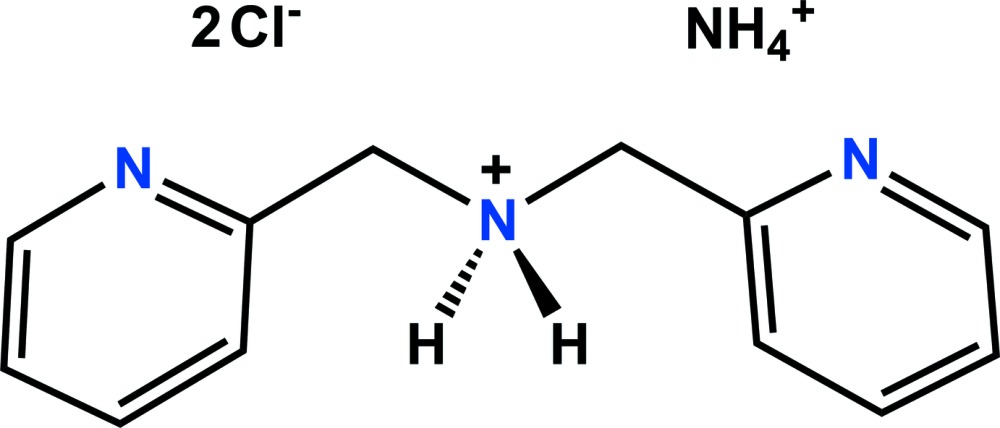


## Experimental   

### Crystal data  

C_12_H_14_N_3_^+^·H_4_N^+^·2(Cl^−^)
*M* *_r_* = 289.20
Orthorhombic, 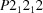

*a* = 8.895 (1) Å
*b* = 17.676 (2) Å
*c* = 4.4360 (5) Å
*V* = 697.47 (14) Å^3^
*Z* = 2
Mo *K*α radiation
μ = 0.45 mm^−1^
*T* = 100 K
0.55 × 0.30 × 0.25 mm

### Data collection  

Bruker APEXII CCD diffractometer
Absorption correction: multi-scan (*SADABS*; Bruker, 2013 ▸) *T*_min_ = 0.605, *T*_max_ = 0.746
4292 measured reflections
2126 independent reflections
2088 reflections with *I* > 2σ(*I*)
*R*_int_ = 0.016

### Refinement  

*R*[*F* ^2^ > 2σ(*F* ^2^)] = 0.025
*wR*(*F* ^2^) = 0.066
*S* = 1.06
2126 reflections
89 parameters
1 restraint
H atoms treated by a mixture of independent and constrained refinement
Δρ_max_ = 0.42 e Å^−3^
Δρ_min_ = −0.17 e Å^−3^
Absolute structure: Flack *x* determined using 748 quotients [(*I* ^+^)−(*I* ^−^)]/[(*I* ^+^)+(*I* ^−^)] (Parsons *et al.*, 2013 ▸)
Absolute structure parameter: 0.04 (2)

### 

Data collection: *APEX2* (Bruker, 2013 ▸); cell refinement: *SAINT* (Bruker, 2013 ▸); data reduction: *SAINT*; program(s) used to solve structure: *SHELXS97* (Sheldrick, 2008 ▸); program(s) used to refine structure: *SHELXL2014* (Sheldrick, 2015 ▸) and *SHELXLE* (Hübschle *et al.*, 2011 ▸); molecular graphics: *OLEX2* (Dolomanov *et al.*, 2009 ▸); software used to prepare material for publication: *publCIF* (Westrip, 2010 ▸).

## Supplementary Material

Crystal structure: contains datablock(s) I. DOI: 10.1107/S2056989015015753/lh5782sup1.cif

Structure factors: contains datablock(s) I. DOI: 10.1107/S2056989015015753/lh5782Isup2.hkl

**Fig. 1.**
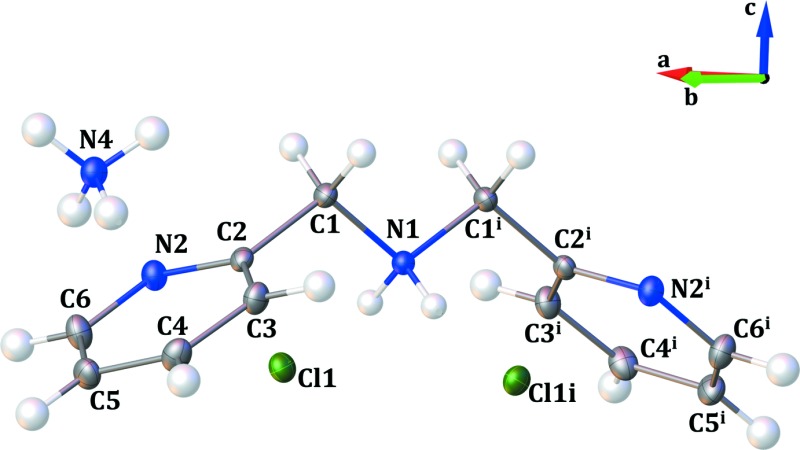
The molecular structure, shown with 50% probability ellipsoids for non-H atoms and circles of arbitrary size for H atoms [symmetry code: (i) -x+2, -y+2, z].

Supporting information file. DOI: 10.1107/S2056989015015753/lh5782Isup3.cml

x y z . DOI: 10.1107/S2056989015015753/lh5782fig1.tif
The mol­ecular structure, shown with 50% probability ellipsoids for non-H atoms and circles of arbitrary size for H atoms [symmetry code: (i) −*x* + 2, −*y* + 2, *z*].

**Fig. 2.**
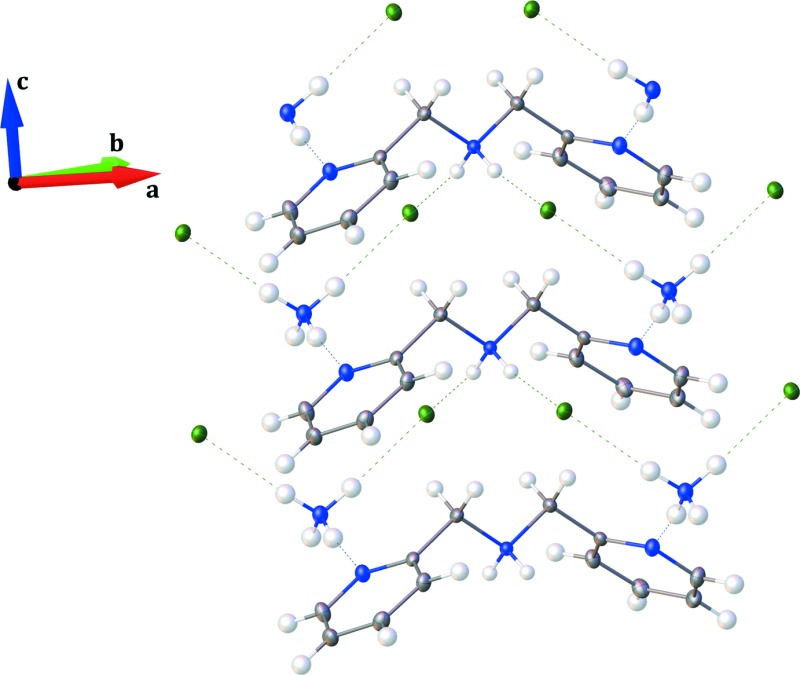
Part of the crystal structure with hydrogen bonds shown as dashed lines.

. DOI: 10.1107/S2056989015015753/lh5782fig2.tif
Part of the crystal structure with hydrogen bonds shown as dashed lines.

CCDC reference: 1420168

Additional supporting information:  crystallographic information; 3D view; checkCIF report

## Figures and Tables

**Table 1:** Hydrogen-bond geometry (, )

*D*H*A*	*D*H	H*A*	*D* *A*	*D*H*A*
C1H1*B*Cl1^i^	0.99	2.80	3.7145(15)	154
C6H6Cl1^ii^	0.95	2.75	3.6410(15)	157
N1H1*C*Cl1^iii^	0.91	2.23	3.1239(9)	168
N1H1*D*Cl1^iv^	0.91	2.23	3.1239(9)	168
N4H4*A*N2	0.90(2)	2.09(2)	2.9748(15)	167(2)
N4H4*B*Cl1	0.93(2)	2.32(2)	3.2362(12)	170(2)

Symmetry codes: (i) 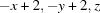; (ii) 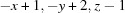; (iii) 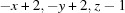; (iv) 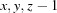.

## supplementary crystallographic information

### S1. Chemical context

Atom transfer Radical Addition (ATRA) reactions involve the formation of carbon-carbon bonds through the addition of saturated poly-halogenated hydro­carbons to alkenes (Eckenhoff & Pintauer, 2010). First reported by Kharasch in the 1940s (Kharasch *et al.*, 1945), the reaction incorporates halogen group functionalities within products; which can then be used as starting reagents in further functionalization reactions (Iqbal *et al.*, 1994). Subsequently, ATRA has emerged as some of the most atom economical methods for simultaneously forming C–C and C–X bonds; leading to the production of more attractive molecules with well-defined compositions, architectures, and functionalities (Braunecker & Matyjaszewski , 2007). Structural studies suggest that the type of ligand used in atom transfer radical reactions significantly influence the behavior of catalyst generated due to different steric and electronic inter­actions with the metal center (Matyjaszewski *et al.*, 2001). Copper complexes made with tetra­dentate nitro­gen-based ligands such as 1,4,8,11-tetra­aza-1,4,8,11-tetra­methyl­cyclo­tetra­decane (Me_6_CYCLAM), *tris*(2-pyridyl­methyl)­amine (TPMA), *tris*(2-(di­methyl­amino)­ethyl)­amine (Me_6_TREN), and *bis*(2-pyridyl­methyl) amine (BPMA) are currently some of the most active multi-dentate structures used in atom transfer radical reactions (Tang *et al.*, 2008). Given the significance of these ligands, we present the crystal structure of a protonated *bis*(2-pyridyl­methyl)­amine (BPMA) salt.

### S2. Structural commentary

The molecular structure of the title compound is shown in Fig 1. The bis­[(pyridin-2-yl)methyl]­ammonium and ammonium cations both lie across a twofold rotation axis. The dihedral angles between the pyridine rings is 68.43 (8)°. This is in contrast to the values of the dihedral anlges in bis­(2-pyridyl­methyl)­ammonium bromide and bis­(2-pyridyl­methyl)­ammonium iodide (Junk *et al.*, 2006) which are 38.47 (13) and 5.17 (9)°, respectively. In the crystal, N—H···N and N—H···Cl hydrogen bonds link the components of the structure forming a two-dimensional network parallel to (010) (Fig. 2). In addition, weak C—H···Cl hydrogen bonds exist within the two-dimensional network.

### S3. Synthesis and crystallization

*Bis*(2-pyridyl­methyl)­amine salt (BPMA) was synthesized and purified following literature procedures (Carvalho *et al.*, 2006) and the reaction scheme is shown in Fig. 3. A 500 mL round bottom flask was filled with 100 mL of methanol then 2-pyridine­carboxaldehyde (8.90 mL, 94.0 mmol) added. The flask was placed in an ice bath to cool with the solution mixing. After 15 minutes, 2-pyridyl­methyl­amine (9.70 mL, 94.0 mmol) was added to give a dull yellow colored solution. Flask was removed from ice bath and mixture allowed to react at room temperature for 1 hour to give a red colored solution. The flask was placed back in an ice bath and sodium borohydride (3.500 g, 94.0 mmol) was added in small amounts to prevent foaming. After this addition, the flask was removed from the ice bath and the mixture left to stir overnight. Concentrated hydro­chloric acid was added to the mixture drop-wise until a pH of 4 was attained producing an orange mixture. An extraction was performed on the mixture in a separatory funnel with di­chloro­methane until the organic phase became colorless. The aqueous phase was separated and its pH adjusted to 10 with Na_2_CO_3_. A second extraction was performed with di­chloro­methane on this mixture and the organic layer isolated and dried using MgSO_4_. Solvent was removed to produce the desired ligand as a dark-brown colored oil (14.910 g, 80%). ^1^H NMR (CDCl_3_, 400 MHz): δ3.48 (s, 1H), δ4.01 (s, 4H), δ7.14 (t, *J* = 7.6 Hz, 2H), δ 7.34 (d, *J* = 7.6 Hz, 2H), δ 7.63 (t, *J* = 7.6 Hz, 2H), δ 8.53 (d, *J* = 4.8 Hz, 2H). ^13^C NMR (CDCl_3_, 400 MHz): δ 156.67, 149.20, 136.74, 122.73, 122.48, 53.25. FT—IR (liquid) *v* (cm^-1^): 3283 (b), 3003 (m), 2818 (b), 1587 (s), 1566 (m), 1471 (s), 1429 (s), 1356 (b). Colorless single crystals suitable for X-Ray analysis were obtained from slow cooling of BPMA ligand in the refrigerator.

### S4. Refinement

All H atoms, except for those of the ammonium cation, were placed in calculated positions and refined in a riding-model approximation, with C—H = 0.95 - 0.99 Å, N—H = 0.91 Å and U_iso_(H) = 1.2U_eq_(C,N). The two unique H atoms of the ammonium cation were refined indpendently with isotropic displacement parameters.

### Crystal data

**Table:**

C_12_H_14_N_3_^+^·H_4_N^+^·2(Cl^−^)	*D*_x_ = 1.377 Mg m^−^^3^
*M**_r_* = 289.20	Mo *K*α radiation, λ = 0.71073 Å
Orthorhombic, *P*2_1_2_1_2	Cell parameters from 3559 reflections
*a* = 8.895 (1) Å	θ = 2.3–31.8°
*b* = 17.676 (2) Å	µ = 0.45 mm^−^^1^
*c* = 4.4360 (5) Å	*T* = 100 K
*V* = 697.47 (14) Å^3^	Rod, colourless
*Z* = 2	0.55 × 0.30 × 0.25 mm
*F*(000) = 304	

### Data collection

**Table:**

Bruker APEXII CCD diffractometer	2126 independent reflections
Radiation source: fine focus sealed tube	2088 reflections with *I* > 2σ(*I*)
Graphite monochromator	*R*_int_ = 0.016
ω and φ scans	θ_max_ = 31.9°, θ_min_ = 2.6°
Absorption correction: multi-scan (*SADABS*; Bruker, 2013)	*h* = −12→12
*T*_min_ = 0.605, *T*_max_ = 0.746	*k* = −25→20
4292 measured reflections	*l* = −6→5

### Refinement

**Table:**

Refinement on *F*^2^	Secondary atom site location: difference Fourier map
Least-squares matrix: full	Hydrogen site location: mixed
*R*[*F*^2^ > 2σ(*F*^2^)] = 0.025	H atoms treated by a mixture of independent and constrained refinement
*wR*(*F*^2^) = 0.066	*w* = 1/[σ^2^(*F*_o_^2^) + (0.0393*P*)^2^ + 0.1504*P*] where *P* = (*F*_o_^2^ + 2*F*_c_^2^)/3
*S* = 1.06	(Δ/σ)_max_ = 0.001
2126 reflections	Δρ_max_ = 0.42 e Å^−^^3^
89 parameters	Δρ_min_ = −0.17 e Å^−^^3^
1 restraint	Absolute structure: Flack *x* determined using 748 quotients [(*I*^+^)-(*I*^-^)]/[(*I*^+^)+(*I*^-^)] (Parsons *et al.*, 2013)
Primary atom site location: structure-invariant direct methods	Absolute structure parameter: 0.04 (2)

### Special details

**Table:**

Geometry. All e.s.d.'s (except the e.s.d. in the dihedral angle between two l.s. planes) are estimated using the full covariance matrix. The cell e.s.d.'s are taken into account individually in the estimation of e.s.d.'s in distances, angles and torsion angles; correlations between e.s.d.'s in cell parameters are only used when they are defined by crystal symmetry. An approximate (isotropic) treatment of cell e.s.d.'s is used for estimating e.s.d.'s involving l.s. planes.

### Fractional atomic coordinates and isotropic or equivalent isotropic displacement parameters (Å^2^)

**Table:**

	*x*	*y*	*z*	*U*_iso_*/*U*_eq_	Occ. (<1)
C1	0.92866 (15)	0.93964 (7)	1.2090 (3)	0.0121 (2)	
H1A	0.8529	0.9629	1.3436	0.015*	
H1B	1.0064	0.9156	1.3367	0.015*	
C2	0.85420 (16)	0.88021 (7)	1.0170 (3)	0.0110 (2)	
C3	0.93696 (16)	0.81886 (8)	0.9096 (4)	0.0149 (3)	
H3	1.0412	0.8146	0.9525	0.018*	
C4	0.86432 (17)	0.76416 (8)	0.7391 (4)	0.0169 (3)	
H4	0.9174	0.7210	0.6683	0.020*	
C5	0.71328 (17)	0.77338 (8)	0.6736 (4)	0.0164 (3)	
H5	0.6615	0.7375	0.5526	0.020*	
C6	0.63891 (16)	0.83632 (8)	0.7888 (4)	0.0168 (3)	
H6	0.5351	0.8424	0.7442	0.020*	
N1	1.0000	1.0000	1.0188 (4)	0.0103 (3)	
H1C	1.0711	0.9787	0.8982	0.012*	0.5
H1D	0.9289	1.0213	0.8982	0.012*	0.5
N2	0.70678 (14)	0.88881 (7)	0.9597 (3)	0.0134 (2)	
Cl1	0.73444 (4)	1.08251 (2)	1.69586 (8)	0.01425 (9)	
N4	0.5000	1.0000	1.2452 (4)	0.0142 (3)	
H4A	0.558 (2)	0.9696 (11)	1.131 (5)	0.021*	
H4B	0.563 (2)	1.0295 (11)	1.363 (5)	0.021*	

### Atomic displacement parameters (Å^2^)

**Table:**

	*U*^11^	*U*^22^	*U*^33^	*U*^12^	*U*^13^	*U*^23^
C1	0.0123 (5)	0.0129 (5)	0.0112 (5)	−0.0025 (4)	0.0005 (5)	0.0010 (5)
C2	0.0108 (6)	0.0111 (5)	0.0111 (5)	−0.0015 (4)	0.0004 (5)	0.0024 (5)
C3	0.0109 (6)	0.0145 (6)	0.0194 (6)	0.0008 (5)	0.0020 (5)	−0.0002 (5)
C4	0.0163 (6)	0.0136 (5)	0.0208 (7)	0.0005 (5)	0.0044 (6)	−0.0024 (5)
C5	0.0165 (6)	0.0151 (5)	0.0176 (6)	−0.0034 (5)	−0.0001 (6)	−0.0038 (5)
C6	0.0126 (6)	0.0152 (6)	0.0225 (7)	−0.0001 (5)	−0.0036 (6)	−0.0016 (6)
N1	0.0099 (7)	0.0105 (6)	0.0104 (7)	−0.0006 (6)	0.000	0.000
N2	0.0118 (5)	0.0118 (4)	0.0167 (6)	0.0002 (4)	−0.0006 (4)	−0.0007 (4)
Cl1	0.01141 (14)	0.01526 (14)	0.01608 (15)	0.00146 (10)	−0.00198 (11)	0.00017 (11)
N4	0.0120 (7)	0.0136 (7)	0.0170 (9)	0.0008 (6)	0.000	0.000

### Geometric parameters (Å, º)

**Table:**

C1—N1	1.5010 (16)	C5—C6	1.392 (2)
C1—C2	1.5060 (19)	C5—H5	0.9500
C1—H1A	0.9900	C6—N2	1.3418 (19)
C1—H1B	0.9900	C6—H6	0.9500
C2—N2	1.3442 (18)	N1—C1^i^	1.5010 (16)
C2—C3	1.3944 (19)	N1—H1C	0.9100
C3—C4	1.387 (2)	N1—H1D	0.9100
C3—H3	0.9500	N4—H4A	0.898 (18)
C4—C5	1.384 (2)	N4—H4B	0.926 (18)
C4—H4	0.9500		
			
N1—C1—C2	111.33 (12)	C4—C5—C6	118.59 (14)
N1—C1—H1A	109.4	C4—C5—H5	120.7
C2—C1—H1A	109.4	C6—C5—H5	120.7
N1—C1—H1B	109.4	N2—C6—C5	123.12 (13)
C2—C1—H1B	109.4	N2—C6—H6	118.4
H1A—C1—H1B	108.0	C5—C6—H6	118.4
N2—C2—C3	122.57 (13)	C1^i^—N1—C1	111.59 (15)
N2—C2—C1	117.19 (12)	C1^i^—N1—H1C	109.3
C3—C2—C1	120.23 (12)	C1—N1—H1C	109.3
C4—C3—C2	118.84 (13)	C1^i^—N1—H1D	109.3
C4—C3—H3	120.6	C1—N1—H1D	109.3
C2—C3—H3	120.6	H1C—N1—H1D	108.0
C5—C4—C3	118.98 (13)	C6—N2—C2	117.87 (12)
C5—C4—H4	120.5	H4A—N4—H4B	108.0 (19)
C3—C4—H4	120.5		
			
N1—C1—C2—N2	−94.13 (14)	C4—C5—C6—N2	0.3 (2)
N1—C1—C2—C3	86.76 (15)	C2—C1—N1—C1^i^	178.68 (13)
N2—C2—C3—C4	−0.5 (2)	C5—C6—N2—C2	1.1 (2)
C1—C2—C3—C4	178.53 (13)	C3—C2—N2—C6	−0.9 (2)
C2—C3—C4—C5	1.9 (2)	C1—C2—N2—C6	179.97 (13)
C3—C4—C5—C6	−1.7 (2)		

Symmetry code: (i) −*x*+2, −*y*+2, *z*.

### Hydrogen-bond geometry (Å, º)

**Table:**

*D*—H···*A*	*D*—H	H···*A*	*D*···*A*	*D*—H···*A*
C1—H1*B*···Cl1^i^	0.99	2.80	3.7145 (15)	154
C6—H6···Cl1^ii^	0.95	2.75	3.6410 (15)	157
N1—H1*C*···Cl1^iii^	0.91	2.23	3.1239 (9)	168
N1—H1*D*···Cl1^iv^	0.91	2.23	3.1239 (9)	168
N4—H4*A*···N2	0.90 (2)	2.09 (2)	2.9748 (15)	167 (2)
N4—H4*B*···Cl1	0.93 (2)	2.32 (2)	3.2362 (12)	170 (2)

Symmetry codes: (i) −*x*+2, −*y*+2, *z*; (ii) −*x*+1, −*y*+2, *z*−1; (iii) −*x*+2, −*y*+2, *z*−1; (iv) *x*, *y*, *z*−1.
