# Supplementary figures and images for: Crystal structure of ammonium bis­[(pyridin-2-yl)meth­yl]ammonium dichloride
Source: Acta Crystallogr E Crystallogr Commun. 2015 Aug 29;71(Pt 9):o692–3. doi: 10.1107/S2056989015015753 (PMC4555372; doi:10.1107/S2056989015015753)

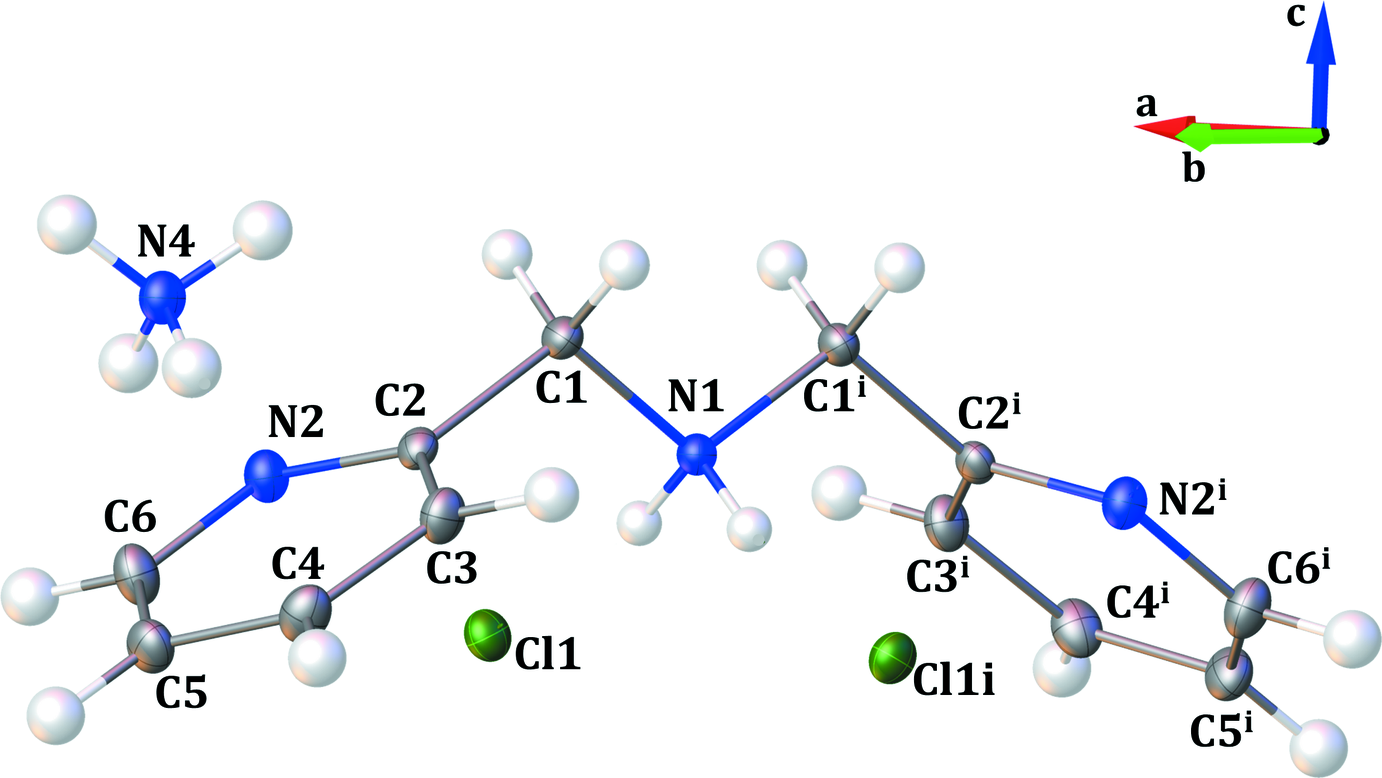

Supplement: Supplementary file 4 [file e-71-0o692-fig1.tif]

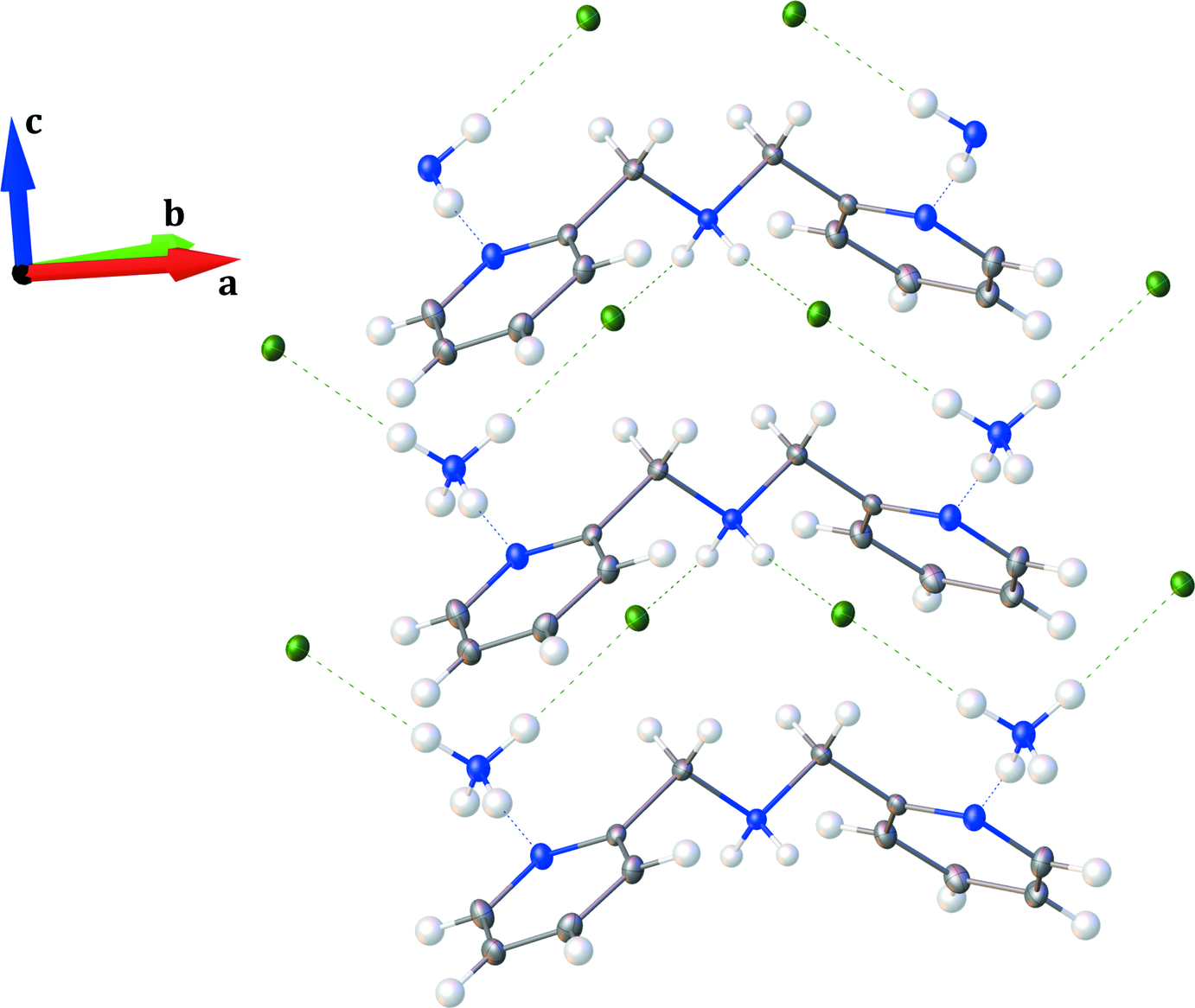

Supplement: Supplementary file 5 [file e-71-0o692-fig2.tif]
